# Performance of the nasal photoplethysmographic index as an analgesic index during surgery under general anaesthesia

**DOI:** 10.1038/s41598-020-64033-0

**Published:** 2020-04-28

**Authors:** Chanki Park, Min-Ho Yang, Bohyun Choi, Bokyung Jeon, Yong-Hun Lee, Hangsik Shin, Boreom Lee, Byung-Moon Choi, Gyu-Jeong Noh

**Affiliations:** 1 Center for Bionics, Korea Institute of Science and Technology, Seoul, South Korea; 2 University of Ulsan College of Medicine, Seoul, South Korea; 3 Department of Nursing, Graduate School and College of Nursing Science, KyungHee University, Seoul, Korea; 4 Department of Anaesthesiology and Pain Medicine, Asan Medical Center, University of Ulsan College of Medicine, Seoul, South Korea; 5 Department of Anaesthesiology and Pain Medicine, Asan Medical Center, University of Ulsan College of Medicine, Seoul, South Korea; 6 Department of Biomedical Engineering, Chonnam National University, Yeosu, South Korea; 7 Department of Biomedical Science and Engineering, Institute of Integrated Technology, Gwangju Institute of Science and Technology, Gwangju, South Korea; 8 Department of Anaesthesiology and Pain Medicine, Asan Medical Center, University of Ulsan College of Medicine, Seoul, South Korea; 9 Department of Anesthesiology and Pain Medicine and Department of Clinical Pharmacology and Therapeutics, Asan Medical Center, University of Ulsan College of Medicine, Seoul, South Korea

**Keywords:** Health care, Medical research

## Abstract

In a previous study, we developed a new analgesic index using nasal photoplethysmography (nasal photoplethysmographic index, NPI) and showed that the NPI was superior to the surgical pleth index (SPI) in distinguishing pain above numerical rating scale 3. Because the NPI was developed using data obtained from conscious patients with pain, we evaluated the performance of NPI in comparison with the SPI and the analgesia nociception index (ANI) in patients under general anaesthesia with target-controlled infusion of propofol and remifentanil. The time of nociception occurrence was defined as when the signs of inadequate anaesthesia occurred. The median values of NPI, SPI, and ANI for 1 minute from the time of the sign of inadequate anaesthesia were determined as the value of each analgesic index that represents inadequate anaesthesia. The time of no nociception was determined as 2 minutes before the onset of skin incision, and the median value for 1 minute from that time was defined as the baseline value. In total, 81 patients were included in the analysis. NPI showed good performance in distinguishing inadequate anaesthesia during propofol-remifentanil based general anaesthesia. NPI had the highest value in terms of area under the receiver operating characteristic curve, albeit without statistical significance (NPI: 0.733, SPI: 0.722, ANI: 0.668). The coefficient of variations of baseline values of NPI, SPI, and ANI were 27.5, 47.2, and 26.1, respectively. Thus, the NPI was effective for detecting inadequate anaesthesia, showing similar performance with both indices and less baseline inter-individual variability than the SPI.

## Introduction

In order to objectively evaluate the degree of nociception during general anaesthesia, commercialized analgesic indexes have been developed^1–5^, among which the surgical pleth index (SPI; GE Healthcare, Milwaukee, WI, USA) and the analgesia nociception index (ANI; PhysioDoloris, MetroDoloris, Loos, France) are mostly widely used and studied. ANI, which is calculated from the heart rate variability analysis, is not regarded to have significant clinical benefit. SPI appears to reflect the noxious stimuli during operation, but it is not clear whether SPI provides actual clinical benefit^4,6^. Also, one study showed that SPI was not useful for assessing pain in conscious patients^7^.

We developed the nasal photoplethysmographic index (NPI), which is calculated from diastolic peak point variation (*Dia*_*var*_) and heart beat interval variation (*HBI*_*var*_); the NPI is the first dynamic index to objectively evaluate pain, and is superior to SPI in distinguishing pain above NRS 3^8^. However, because NPI was developed using photoplethysmographic data obtained from conscious patients with pain (numerical rating scale ≥ 3), the performance of the NPI during general anaesthesia needs to be assessed. We thus evaluated the performance of the NPI as an analgesic index in comparison with SPI and ANI in patients under general anaesthesia with target-controlled infusion of propofol and remifentanil.

## Methods

### Patient population

The study protocol was approved by the Institutional Review Board of Asan Medical Center (#2017–0095, Chairperson: Prof Moo-Song Lee, Approval date: 31 January, 2017) and registered on an international clinical trials registry platform (http://cris.nih.go.kr; KCT0002723). All methods were performed in accordance with the relevant guidelines and regulations of the institution. Eighty-nine patients (American Society of Anesthesiologists Physical Status 1, 2 or 3) between the ages of 20 and 80 who were scheduled to undergo elective surgery were included in this observational study. Written informed consent was obtained from all patients. Exclusion criteria were as follows: clinically significant impairment of cardiovascular, hepatic, or renal function; history of cardiac arrhythmia; use of medication that might affect autonomic function; clinically significant laboratory findings; and evidence of pregnancy.

### Procedure and data acquisition

All patients fasted beginning at midnight on the day of surgery, and no premedication was administered. In the operating theatre, the patients were monitored for their heart activity by using electrocardiography, end-tidal carbon dioxide partial pressure, and non-invasive blood pressure. A specially designed sensor was placed between the columella and nasal septum to acquire nasal photoplethysmography^8^. NPI score was calculated by retrospectively analyzing the nasal photoplethysmography wave data. A reusable SPI sensor was placed on the index finger of the arm that was not used for blood pressure measurement. A disposable ANI sensor was placed on the chest according to the manufacturer’s manual. SPI and ANI values were recorded at 10-s and 1-s intervals on a laptop computer for offline analysis, respectively. General anaesthesia was performed by administering propofol and remifentanil by a target effect-site concentration-controlled infusion using the Schnider and Minto models^9,10^. Target effect-site concentrations (*Ces*) of propofol were titrated to maintain bispectral index (BIS, Covidien, Boulder, CO, USA) values of less than 60 during induction and maintenance of anaesthesia^8^. Target *Ces* of remifentanil were adjusted to maintain stable haemodynamics (i.e., systolic blood pressure >80 mmHg; heart rate >45 beats/min)^8^. Tracheal intubation was facilitated by administration of rocuronium at 0.6 mg/kg. Patients were then ventilated with oxygen in air (1:2), and the ventilation rate was adjusted to maintain an end-tidal carbon dioxide partial pressure of 4.7–6.0 KPa. All patients received a continuous infusion of Ringer’s lactate solution (8–10 ml/kg/h) during the maintenance of anaesthesia.

### Signs of inadequate anaesthesia

When propofol and remifentanil are administered concomitantly during general anaesthesia, it is difficult to clearly determine whether the patient is feeling nociception; therefore, we defined the time of nociception occurrence as the occurrence of the signs of inadequate anaesthesia, which included the following^11^: 1) increase in systolic blood pressure of more than 15 mmHg above the respective normal value for each patient (the lowest pressure measured at hospital admission); 2) heart rate greater than 90 beats/min in the absence of hypovolemia; 3) autonomic signs such as sweating, flushing, or lacrimation; and 4) somatic responses such as movements, swallowing, coughing, grimacing, or eye movement. The median values of NPI, SPI, and ANI during 1 minute from the time of the sign of inadequate anaesthesia were determined as the value of each analgesic index that represents inadequate anaesthesia. After obtaining these data, the target concentration of remifentanil was increased by 2–3 ng/ml according to conventional anaesthesia methods. Once inadequate anaesthesia occurred, the frequency of occurrence was considered to be 1 until complete resolution. The time of no nociception was determined as 2 minutes before the onset of skin incision, and the median value for 1 minute from that time was used as the baseline value. The target concentration of remifentanil at the time of skin incision was maintained at ≥7 ng/ml and maintained within the range of 7–20 ng/ml during the operation.

### Statistical anaylsis

The predictive value (sensitivity and specificity) of the cut-offs of analgesic indices for detecting inadequate anaesthesia was calculated with the receiver operating characteristic (ROC) curve^8^. The differences in the ROC curves were calculated with the MedCalc Statistical Software (version 13.3.1, MedCalc Software bvba, Ostend, Belgium). Prediction probability (Pk) was assessed as described by Smith and colleagues^12^. Prediction probability values were calculated using Somers’ d cross-tabulation statistic on SPSS, which were then transformed from the –1 to 1 scale of Somers’ d to the 0 to 1 scale of prediction probability, as prediction probability = 1 − (1 −│Somers’ d│)/2. The NPI, SPI, and ANI were set as the dependent variables for the Somers’ d cross-tabulation statistic, and the response (0: adequate anaesthesia, 1: inadequate anaesthesia) was set as the independent variable. Prediction probabilities were calculated using the full measurement set. The standard error (SE) of each prediction probability was calculated as (SE of Somers’ d)/2. To evaluate inter-individual baseline variability, coefficient of variation (CV) was calculated by dividing the standard deviation by mean and multiplying by 100. Statistical analyses were conducted using R (version 3.6.0; R Foundation for Statistical Computing, Vienna, Austria), GraphPad Prism (version 8.1.2, Graph Pad Software, San Francisco, California, USA), or IBM SPSS (version 22.0, SPSS Inc., Chicago, IL, USA). Data are expressed as mean ± standard deviation for normally distributed continuous variables, median (25–75%) for non-normally distributed continuous variables, or count and percentage for categorical variables. *P* values < 0.05 were considered to be statistically significant.

## Results

**Figure 1:**
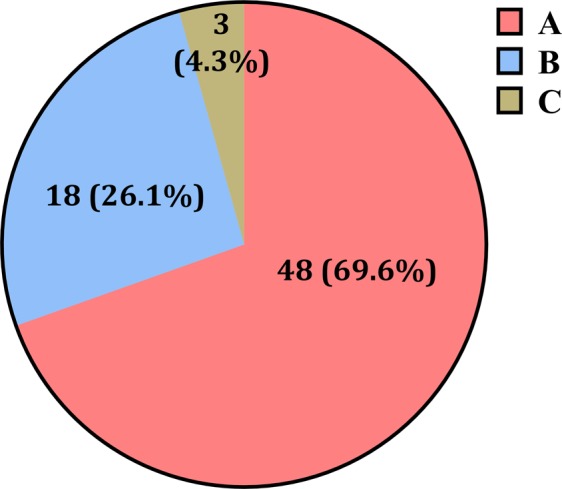
Proportion of the occurrence of signs for inadequate anesthesia. (**A**) increase in systolic blood pressure of more than 15 mmHg above the respective normal value in each patient (the lowest pressure measured at hospital admission), (**B**) heart rate greater than 90 beats/min in the absence of hypovolemia, (**C**) autonomic signs such as sweating, flushing, or lacrimation. When the increases in systolic blood pressure and heart rate occurred at the same time, the higher rate of increase was selected.

A total of 95 patients were screened, of whom 6 were excluded due to violations of the inclusion criteria. A total of 89 patients were enrolled, and 8 patients were dropped out because of withdrawal of consent (n = 2), change of surgery schedule (n = 4), and failure of data storage (n = 2). Hence, 81 patients were included in the analysis. The characteristics of these patients are summarised in Table 1. A total of 69 signs of inappropriate anesthesia were observed in 43 patients and not in the remaining 38 patients. Thus, 81 baseline values and 69 inappropriate anesthesia values were used for the analysis. Proportion of the occurrence of signs for inadequate anesthesia is depicted in Fig. 1. In most cases, systolic blood pressure and/or heart rate were increased. The weighting coefficients of two variables for NPI were changed to better distinguish the presence of inappropriate anaesthesia during surgery, and the values were as follows.

$$NPI=453.34\times Di{a}_{\mathrm{var}}-3.21\times HB{I}_{\mathrm{var}}+6.09,$$

**Table 1:** Characteristics of the patients. Values are mean (SD) or counts.

	Patients (*n* = 81)
Male/Female	48/33
Age, yr	55.5 (10.1)
Height, cm	164.0 (7.8)
Weight, kg	63.2 (9.7)
ASA 1/2	33/48
Duration of anaesthesia, min	178.0 (56.5)
Operation	
ST	62
CRS	14
HBP	5

**Figure 2:**
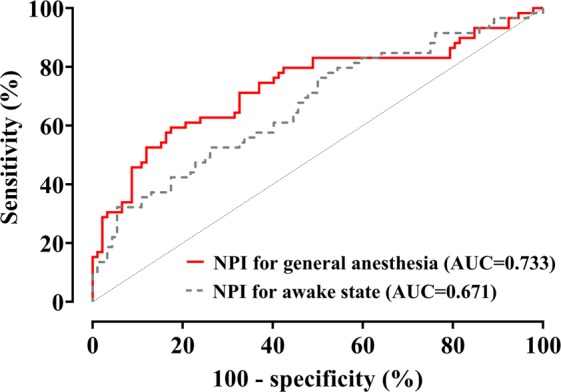
Receiver-operating characteristic curves of the Nasal Photoplethysmography Indices (NPI). NPI for general anesthesia: $NPI=453.34\times Di{a}_{\mathrm{var}}-3.21\times HB{I}_{\mathrm{var}}+6.09$, NPI for awake state: $NPI=462.43\times Di{a}_{\mathrm{var}}-683.11\times HB{I}_{\mathrm{var}}+35.55$. This equation is quoted from a previous study^8^.

**Figure 3:**
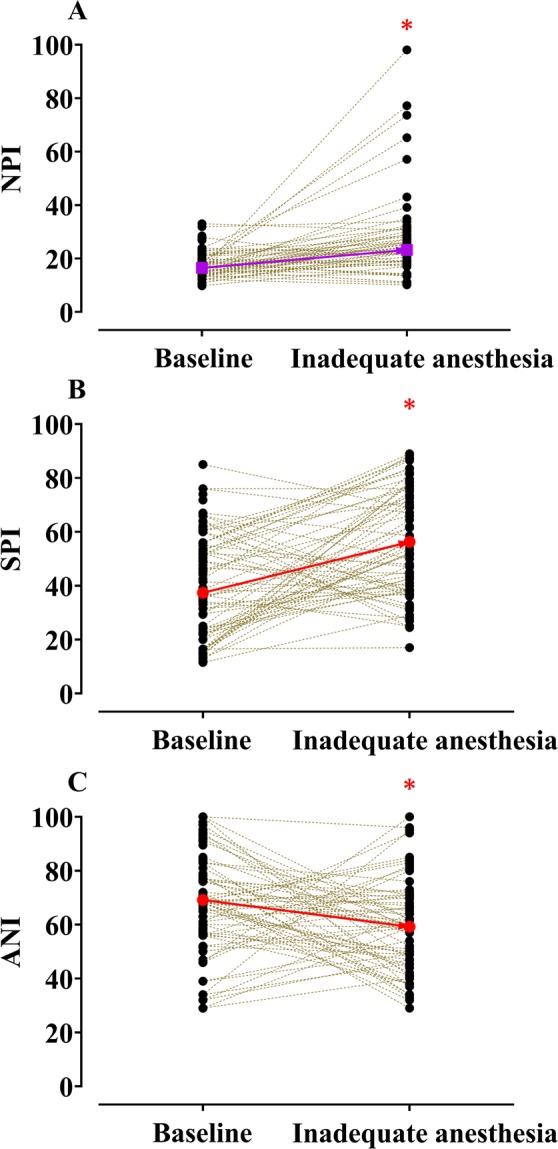
Individual changes in Nasal Photoplethysmography Index (NPI, **A**), Surgical Pleth Index (SPI, **B**), and Antinociception Index (ANI, **C**) without and with inadequate anaesthesia. *P < 0.05 *vs*. baseline. Purple squares and red circles indicate median and mean values, respectively. NPI values were calculated using the intraoperative NPI equation.

ASA, American Society of Anesthesiologists physical status; ST, stomach surgery including distal or total gastrectomy; CRS, colorectal surgery including right hemicolectomy, anterior resection, Hartmann’s operation, and ileostomy takedown; HBP, hepatobiliary surgery including right hepatectomy, left medial segmentectomy, and total pancreatectomy.

where *Dia*_*var*_ and *HBI*_*var*_ indicate diastolic peak point variation and heartbeat interval variation, respectively. ROC curves of the NPI calculated by different coefficients are shown in Fig. 2. The AUC was increased by changing the coefficients of NPI variables. Individual changes in NPI, SPI, and ANI without and with inadequate anaesthesia are presented in Fig. 3. In case of inadequate anaesthesia, the median (25–75%) NPI value increased significantly (baseline: 16.5 (14.4–20.3) vs. inadequate anaesthesia: 23.2 (18.3–29.0), *P* < 0.001 in signed-rank test). The mean (SD) effect-site concentrations of propofol and remifentanil at the time of inadequate anaesthesia sign were 2.6 (0.2) μg/ml and 9.6 (2.8) ng/ml, respectively. The AUC and cut-off values for detecting inadequate anesthesia in NPI, SPI, and ANI are shown in Table 2. The NPI was not statistically superior to the other indices (NPI *vs*. ANI: *P* = 0.3196, ANI *vs*. SPI: *P* = 0.4037, NPI *vs*. SPI: *P* = 0.8527). Prediction probability values (SE, 95% CI) of NPI, SPI, and ANI calculated from 69 pairs of data were 0.5618 (0.0128, 0.5368–0.5868), 0.5570 (0.0085, 0.5403–0.5736), and 0.5317 (0.0065, 0.5189–0.5445), respectively. The coefficient of variations of baseline values of NPI, SPI, and ANI were 27.5, 47.2, and 26.1, respectively, indicating that NPI and ANI had smaller values of inter-individual variability than did SPI.

**Table 2:** The areas under the receiver-operating characteristic curves (AUC) and cut-off values for detecting inadequate anesthesia in the Nasal Photoplethysmography Index (NPI), Surgical Plethysmographic Index (SPI), and Analgesia Nociception Index (ANI) in surgical patients.

	NPI	SPI	ANI
AUC (95% CI)	0.733 (0.655–0.802)	0.722 (0.643–0.791)	0.668 (0.586–0.742)
*P* value	<0.0001	<0.0001	0.0003
Cut-off value (sensitivity %, specificity %)	21.8 (59.3, 82.6)	35.5 (86.8, 45.7)	67 (71.2, 60.4)

NPI values were calculated using the intraoperative NPI equation.

## Discussion

The NPI, which is calculated from the diastolic peak point variation and the heartbeat interval variation, showed good performance in distinguishing inadequate anaesthesia during propofol-remifentanil-based general anaesthesia. NPI had the highest value in terms of area under the ROC curve, albeit without statistical significance. Also, NPI had better baseline stability than did SPI.

To date, several commercialized analgesic indices have been used to evaluate intraoperative nociception—of them, the SPI and ANI have been extensively studied in terms of their performance in patients undergoing general anesthesia^1,2,6^. These two indices could be used as a control for evaluating the performance of the newly developed NPI in patients under general anaesthesia, because their algorithms were developed specifically for use in patients under general anesthesia.

A previous study showed that the NPI clearly distinguishes pain (numerical rating scale ≥ 3) in awake surgical patients with postoperative pain and had better performance than the SPI^8^, and our current study showed similar results in patients undergoing general anaesthesia as well. However, in order to better distinguish inadequate anaesthesia during surgery, the weighting coefficients of the two variables that determine the NPI value needed to be changed from the conscious state. It is difficult to say that the nasal photoplethysmographic wave characteristics of conscious state and general anaesthesia state are the same. First, various anesthetic agents administered during general anaesthesia, including propofol and remifentanil, can affect the amplitude and interval of photoplethsymogram. In fact, the diastolic peak point variation at baseline in patients under general anaesthesia (0.025 [0.011]) was lower than that in conscious patients (0.030 [0.019]). Both propofol and remifentanil can induce bradycardia, which affects the interval of photoplethsymogram. Second, the difference in the respiratory patterns between spontaneous breathing in conscious patients with pain and regular mechanical ventilation in anaesthetised patients may also differentially affect the photoplethysmographic wave, with a previous study showing that positive pressure ventilation significantly affects the photoplethysmographic waveform^13^. Third, the atmospheric temperatures in the operating theatre may also affect the photoplethysmographic waveform. In general, the temperature in the operating theatre tends to be lower than that in postanaesthesia care units. One study showed that the photoplethysmographic amplitude varies depending on the fluctuation of the skin temperature^14^. It is reasonable that the weight coefficient of the variables must be different to distinguish the pain from the conscious state and the general anaesthesia state. For this reason, the SPI may not be able to properly reflect pain in the conscious state^6^. In the case of ANI, the ability to distinguish pain from conscious state and general anaesthesia state was inferior to other commercial analgesic indexes, which calls the need for improvement in its algorithm.

It is also important to decide on the baseline when there is no nociception during surgery. As mentioned earlier, considering that the respiratory pattern can affect the PPG wave, the baseline was set at the time at which the pain was expected to be absent during mechanical ventilation. Thus, in this study, the baseline was set at 2 minutes before skin incision. A previous study showed that SPI responses to noxious stimuli due to endotracheal intubation were restored after approximately 5 minutes^15^. As the mean time from the endotracheal intubation to the skin incision was 21.5 minutes in this study, the noxious stimulation by endotracheal intubation is not likely to have significantly affected the baseline time point.

According to a previous study on the performance of the SPI in awake patients^8^, the threshold of the SPI for detecting pain was 43.7 in the awake state and 35.5 in the general anesthesia state. The cut-off value of SPI presented in this study was lower than the manufacturer’s recommendation. Although the SPI values of less than 50 are regarded as acceptable for intraoperative analgesia, the basis for this criterion is relatively lacking^6^. One study also showed that the cut-off value for an ideal SPI was different according to age (children *vs*. adults)^16^. An SPI value lower than the manufacturer’s recommendation for representing sufficient analgesia may potentially improve SPI-guided anaesthesia^6^, and the results of this study are expected to serve as a basis for this argument.

In order to develop an effective analgesic index, it is necessary to have a performance that is closely proportional to the severity of pain. It is also essential to show the stability that presents consistent values in a certain state. Baseline stability is important because current commercialized analgesic indices assess the presence of nociception by absolute values rather than percentage decreases from baseline. In an earlier study, we confirmed that the baseline stability of SPI is low^15^, and the same results were observed in the current study. The reason why NPI has better baseline stability than SPI was likely due to the fact that the NPI variable was normalized for each individual.

The limitations of the current study should be discussed. First, there may be some controversy on using the time of the occurrence of signs of inadequate anaesthesia as the time for nociception occurrence. However, because noxious stimuli may be blunted during general anaesthesia in which propofol and remifentanil are co-administered, nociception occurrence during surgery cannot be objectively determined. Thus, several previous studies have also regarded the sign of inadequate anaesthesia to be the indicator of nociception occurrence^17–19^. Second, whether NPI works well in general anaesthesia using volatile agents is yet to be validated. Some reports showed that the ANI value is affected by the anesthetic method ^20,21^. Sevoflurane-based anaesthesia was significantly higher in the total power of heart rate variability than in the total intravenous anaesthesia at the time of tracheal intubation and maximum operative trauma^22^, and these characteristics were maintained even after tracheal extubation^22^. However, as far as we know, there is no evidence of SPI values changing according to different anaesthetic methods. Since the algorithm of NPI is closer to that of SPI than ANI, NPI can also be expected to work well in general anaesthesia using volatile anesthetics. Additional research is needed to clarify this issue.

In conclusion, the NPI was effective for detecting inadequate anaesthesia during general anaesthesia with propofol and remifentanil. NPI had the highest value in terms of area under the ROC curve, albeit without statistical significance. However, NPI had less baseline interindividual variability than SPI in the absence of nociception.

## Data Availability

The datasets generated during and/or analyzed during the current study are available from the corresponding author on reasonable request
